# Controlling the neuronal balancing act: optical coactivation of excitation and inhibition in neuronal subdomains

**DOI:** 10.1186/1471-2202-14-S1-P348

**Published:** 2013-07-08

**Authors:** Sarah Jarvis, Konstantin Nikolic, Nir Grossman, Simon R Schultz

**Affiliations:** 1 Department of Bioengineering, Imperial College, London SW7 2AZ, UK; 2 Institute of Biomedical Engineering, Department of Electrical and Electronic Engineering, Imperial College, London SW7 2AZ, UK

## 

Optogenetics provides a powerful tool with which to not only observe but also manipulate neuronal activity, due to the possibility to selectively activate spatially targeted subpopulations of neurons. Much focus has been placed on excitatory opsins, such as channelrhodopsin-2 (ChR2) [1], a light-gated cation channel which when illuminated locally depolarizes the neuron membrane and can illicit an action potential (optimal activation wavelength λ = 490nm). Previously, we investigated the effect of illuminating targeting spatially distinct subdomains of the neuron for cortical layer V pyramidal neurons [2]. We demonstrated that whole cell, rather than partial, illumination had the highest efficiency for producing an action potential.

More recently, the possibilities provided by ChR2 to probe the kinetics of action potential generation were further complemented by the development of silencing opsins that hyperpolarize the membrane, including halorhodopsin (NpHR) [3] and Archaerhodopsin T (ArchT) [4]. Both HR and ArchT have been demonstrated *in vivo *to silence activity for populations, but there has been little characterization of how they can be used to modulate action potential generation in individual neurons. As they can be illuminated independently of ChR2 (optimal activation wavelengths λ = 585, 575 for NpHR and ArchT respectively), together these two families of opsins provide a potential window through which to examine the interplay of competing excitatory and inhibitory inputs for differing spatial and temporal patterns of activation. Such characterization has implications for advancing our understanding of the non-linear nature of inhibition, and is also vital for identifying effective protocols for silencing activity - especially while the spatial range and depth of illumination has experimental limitations that prevent deep or large areas being easily accessible. More importantly, it may allow more subtle changes to be made to neuronal function, for instance by altering the transfer function of genetically targeted classes of neurons.

Here, we present the results of study in which we have modeled neurons expressing NpHR and ArchT in NEURON. Using these models, we investigate common illumination strategies and examine the emergence of the subthreshold dynamics by simultaneously illuminating ChR2 and silencing opsins, both for disjoint and identical spatial locations. Finally, we activate silencing opsins while providing excitatory input via illumination of ChR2 in the proximal apical and basal dendrites, mimicking the location of excitatory presynaptic synapses *in vivo*. We demonstrate that it is possible to "reprogramme" the current-to-firing-rate transfer function of a neuron [5], altering both gain and threshold in either direction, by optical neuromodulation using a combination of ChR2 and ArchT or NpHR targeted to appropriate areas of the neuronal morphology. Using these results, we propose a more effective, targeted stimulation protocol for successfully inhibiting neuronal populations in cortical layers.
